# ^99m^Tc-HMPAO SPECT imaging reveals brain hypoperfusion during status epilepticus

**DOI:** 10.1007/s11011-021-00843-z

**Published:** 2021-09-27

**Authors:** Pablo Bascuñana, Bettina J. Wolf, Ina Jahreis, Mirjam Brackhan, Luis García-García, Tobias L. Ross, Frank M. Bengel, Marion Bankstahl, Jens P. Bankstahl

**Affiliations:** 1 Department of Nuclear Medicine, Hannover Medical School, Carl-Neuberg-Str. 1, 30625 Hannover, Germany; 2 Present Address: Department of Neuropathology, University of Oslo and Oslo University Hospital, Oslo, Norway; 3 Department of Pharmacology, Toxicology and Pharmacy, University of Veterinary Medicine, Hannover, Germany; 4 Unidad de Cartografía Cerebral, Instituto Pluridisciplinar, Universidad Complutense de Madrid, Paseo Juan XXIII, 1, 28040 Madrid, Spain; 5 Departamento de Farmacología, Farmacognosia y Botánica, Facultad de Farmacia, Universidad Complutense de Madrid, Plaza Ramón y Cajal s/n, 28040 Madrid, Spain; 6 Hannover Medical School, Institute for Laboratory Animal Science, Hannover, Germany

**Keywords:** Brain perfusion, Pilocarpine, Neuroimaging, Epilepsy

## Abstract

Status epilepticus (SE) is a clinical emergency with high mortality. SE can trigger neuronal death or injury and alteration of neuronal networks resulting in long-term cognitive decline or epilepsy. Among the multiple factors contributing to this damage, imbalance between oxygen and glucose requirements and brain perfusion during SE has been proposed. Herein, we aimed to quantify by neuroimaging the spatiotemporal course of brain perfusion during and after lithium-pilocarpine-induced SE in rats. To this purpose, animals underwent ^99m^Tc-HMPAO SPECT imaging at different time points during and after SE using a small animal SPECT/CT system. ^99m^Tc-HMPAO regional uptake was normalized to the injected dose. In addition, voxel-based statistical parametric mapping was performed. SPECT imaging showed an increase of cortical perfusion before clinical seizure activity onset followed by regional hypo-perfusion starting with the first convulsive seizure and during SE. Twenty-four hours after SE, brain ^99m^Tc-HMPAO uptake was widely decreased. Finally, chronic epileptic animals showed regionally decreased perfusion affecting hippocampus and cortical sub-regions. Despite elevated energy and oxygen requirements, brain hypo-perfusion is present during SE. Our results suggest that insufficient compensation of required blood flow might contribute to neuronal damage and neuroinflammation, and ultimately to chronic epilepsy generated by SE.

## Introduction

Status epilepticus (SE) is a clinical emergency characterized by continuous epileptic seizure activity and is often of sufficient duration to produce irreversible neuronal damage (Walker 2018). SE results from mechanisms leading to seizing activity in combination with failure of the endogenous mechanisms responsible for seizure prevention or termination (Walker 2018). SE can trigger neuronal injury and also alterations of neuronal networks that may result in long-term cognitive decline or epilepsy (Hesdorffer et al. 1998).

Animal models of SE are currently used to study epileptogenesis and are widely applied to evaluate new anti-seizure and antiepileptogenic treatments (Martín and Pozo 2006). Thus, knowledge of the neuropathology of SE is extensive, but mostly focused on its later consequences. Among animal models, the rat lithium-pilocarpine model is one of the most widely used. After pilocarpine-induced SE, rats show neuroinflammation, neuronal loss, reactive gliosis, axonal sprouting, and neurotransmitter imbalance among other alterations, finally leading to chronic epilepsy (Bascuñana et al. 2018; Bascunana et al. 2019; Brackhan et al. 2016).

It has been theorized that the damage induced by SE is caused not only by the excessive neuronal activation directly leading to excitotoxicity, but also due to an imbalance between oxygen and glucose requirements and insufficient brain perfusion during SE (Meletti et al. 2018). An increase on glucose and oxygen requirements not paralleled by an enhance in brain perfusion might lead to hypoxia and reduced energy which triggers disruption of cellular homeostasis and therefore, neurodegeneration (Meletti et al. 2018). In addition, decreased brain perfusion has been correlated to neuronal damage (Pereira de Vasconcelos et al. 2002). Perfusion-weighted magnetic resonance imaging (MRI) may be used to investigate perfusion changes during SE in patients or animal models (Meletti et al. 2018), but needs to be performed under anesthesia to immobilize the subject. However, anesthesia is known to influence brain perfusion (Slupe and Kirsch 2018).

SPECT perfusion imaging using ^99m^Tc-hexa-methyl-propylene-amine-oxime (HMPAO) might be an alternative for investigation of brain perfusion during SE. HMPAO crosses the blood-brain barrier and is converted rapidly from the lipophilic to the hydrophilic state, becoming intracellularly trapped. Once trapped, HMPAO remains stable with little wash-out effect (Tikofsky et al. 1993). Due to its fast kinetics, ^99m^Tc-HMPAO SPECT allows assessment of the brain perfusion state without anesthesia, as the tracer gets trapped in the first minutes after injection (Andersen 1989) and brain retention is very stable. Here, we used ^99m^Tc-HMPAO SPECT imaging to study brain perfusion before, during and after SE in the lithium-pilocarpine rat model.

## Experimental procedures

### Animals

Adult female Sprague-Dawley rats were purchased with 12 weeks of age (n = 44; Envigo, Italy) and pair-housed in ventilated bio-containment units under a 14/10-h light/dark cycle with free access to standard laboratory chow and autoclaved tap water. Animals were allowed to adapt to housing conditions and repetitive handling before starting the experiments. All the experiments were conducted in accordance with European Communities Council Directive 2010/63/EU and were formally approved by the responsible local authority. Data is reported in accordance with the ARRIVE guidelines.

### Status epilepticus induction

SE was induced two weeks after arrival as described previously (Brackhan et al. 2016). Shortly, rats (n = 36) were pre-treated with lithium chloride (127 mg/kg, p.o.) 14–16 h before pilocarpine injection. Methyl-scopolamine (1 mg/kg, i.p.; Sigma-Aldrich, Germany) was administered 30 min before a bolus injection of pilocarpine hydrochloride (30 mg/kg, i.p.; Sigma-Aldrich, Germany), followed by a maximum of 3 injections (10 mg/kg) at 30 min intervals as needed until SE onset (Brackhan et al. 2016). SE was interrupted after 90 min by repeated administration of diazepam (maximum 25 mg/kg, i.p; Ratiopharm). A pilocarpine dose of 34.40 ± 5.83 mg/kg was needed to induce SE without significant differences between groups. Epilepsy stage was confirmed by reporting behavioral seizures happening during daily handling in the animal room. Rats classified as chronic epileptic were scanned at 12 weeks after SE. All scanned animals exhibited at least two generalized spontaneous seizures.

### SPECT imaging

Awake animals were injected intravenously at different time points with 85.7 ± 12.1 MBq ^99m^Tc-HMPAO synthesized using a standard preparation kit (Ceretec, GE Healthcare): (i) baseline (naïve animals; n = 8), (ii) 15 min after the first pilocarpine injection (n = 6), (iii) within seconds after start of the first generalized seizure (n = 8), (iv) 15 min after SE onset (n = 5), (v) 24 h after SE (n = 9), and (vi) in the chronic epileptic stage (12 weeks after SE; n = 5). Animals were anesthetized using isoflurane (1–2% in 100% oxygen) 110 min after radiotracer injection and placed prone in an imaging chamber (Minerve, France). Animals were continuously warmed and monitored for heart and respiration rate, maintaining respiratory rate at 60–80 breaths/min. The SPECT scan was started 120 min after tracer injection with the brain at the center of the field of view using the Explore speCZT camera (GE Healthcare) with a rat 5-pinhole collimator (Trifoil Imaging). Projection data were acquired in step-and-shoot mode with 108 views per pinhole (0.67° increment angle, and 30s per step) followed by a low-dose computed tomography (CT) scan. Energy threshold was set at 60 keV, with a reconstruction window of 125–150 keV for ^99m^Tc. Images were reconstructed using maximum likelihood expectation maximization with 50 iterations to a 156 × 156 × 216 image matrix (0.5 mm pixel size).

### Image analysis

CT images were fused to an MRI template (Schiffer et al. 2006) using PMOD 3.7 software (PMOD Technologies, Switzerland). Subsequently, SPECT images were matched to the template using the corresponding spatial transformation. A region of interest (ROI) atlas (Schwarz et al. 2006) was applied to the co-registered images as previously described (Brackhan et al. 2016). Average total counts for each ROI were divided by the injected dose. Co-registered images were further analyzed by statistical parametric mapping (SPM). Differences in ^99m^Tc-HMPAO uptake between each time point of interest and baseline were analyzed by a two-sample unpaired t-test using SPM12 (UCL, UK) in MATLAB software (MathWorks, USA) setting a significance level threshold of 0.05 (uncorrected for multiple comparisons) and a minimum cluster size of 100 voxels.

### Statistics

Data were analyzed using statistical software (Graphpad Prism 7, La Jolla, CA, USA). Regional differences between time points were analyzed by ANOVA followed by Dunnett’s post hoc test with baseline animals as control group. Differences were considered statistically significant if p < 0.05. Data are shown as mean ± standard deviation (SD).

## Results

**Fig. 1:**
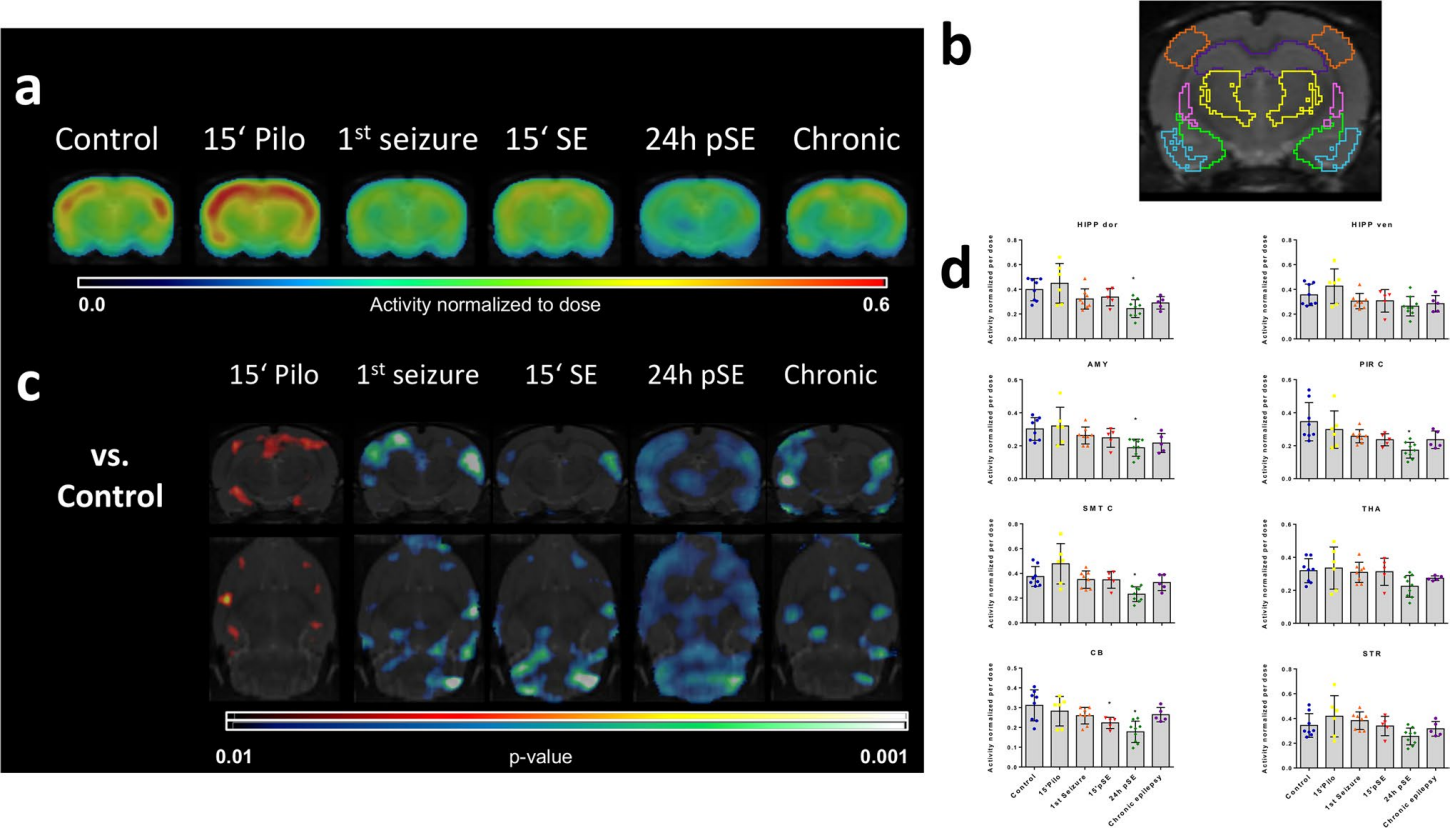
^99m^Tc-HMPAO uptake normalized to the injected dose. (A) Coronal views from the average brain ^99m^Tc-HMPAO uptake images fused to an MRI template for each experimental group. (B) Coronal section of the ROIs used to quantify ^99m^Tc-HMPAO uptake delineated on the MRI template at level Bregma −3.6. (C) SPM t-maps (in coronal and transversal views) showing significantly higher (hot scale) and lower (cold scale) uptake voxels at each time point compared to the baseline group (p < 0.05; minimum cluster size: 100 voxels). (D) Bar graph showing quantification of ^99m^Tc-HMPAO uptake at the different time points in dorsal (HIPP dor) and ventral hippocampus (HIPP ven), amygdala (AMY), piriform cortex (PIR C), somatosensory cortex (SMT C), thalamus (THA), cerebellum (CB) and striatum (STR). Data are shown as mean ± SD. Statistical analysis was performed by ANOVA and Dunnett’s post hoc test. Asterisks indicate significant differences to the baseline group (p < 0.05)

^99m^Tc-HMPAO uptake normalization to the injected dose showed a decrease in the cerebellum during SE (−28%; p = 0.042) using the ROIs atlas analysis (Fig. 1A, B). This analysis method revealed no other significant differences during SE. Twenty-four hours after SE, ^99m^Tc-HMPAO SPECT showed a generalized uptake reduction compared to baseline affecting mainly the dorsal hippocampus (−39%; p = 0.008), cortical regions (e.g. piriform cortex: −50%; p < 0.001), and cerebellum (−43%; p < 0.001). In the chronic epileptic phase, brain perfusion did not differ from baseline values.

On the other hand, SPM analysis showed local alterations in ^99m^Tc-HMPAO uptake of different extent at every studied time point (Fig. 1C). Parametric mapping showed an increased ^99m^Tc-HMPAO uptake in localized cortical regions 15 min after the first pilocarpine injection, i.e. before the first seizure. Already during the first seizure, we found a significant decrease of ^99m^Tc-HMPAO uptake in cortical sub-regions. This decrease in ^99m^Tc-HMPAO was also present during the SE, mainly affecting cortical and cerebellar regions. SPM analysis confirmed the ROIs results, showing a generalized uptake reduction at 24 h after SE. It also revealed decreased ^99m^Tc-HMPAO uptake in cortical and hippocampal sub-regions in chronic epileptic rats.

## Discussion

Here, we investigated brain perfusion during and after SE induced by lithium-pilocarpine in rats by brain imaging without the influence of anesthesia. After initial hyper-perfusion before SE onset, regional perfusion deficits during SE and acute epileptogenesis, as well as during chronic epilepsy became apparent.

A decrease in brain perfusion has previously been shown to correlate with later neuronal damage in adult animals (Pereira de Vasconcelos et al. 2002). Metabolic requirements during SE are increased due to higher glucose and oxygen demand (Wasterlain et al. 1993). We have previously shown an increased glucose consumption at 4 and 24 h after SE in the pilocarpine rat model (Bascuñana et al. 2018). However, ^99m^Tc-HMPAO SPECT imaging showed region-specific perfusion reduction during SE and whole-brain reduction at 24 h post SE despite an initial increase of cortical perfusion after pilocarpine injection. This divergence between metabolic requirements and perfusion during the SE may induce cell stress and lead to neuroinflammation and neuronal death already described in this animal model (Brackhan et al. 2016). On the other hand, the initial increase of perfusion in cortical regions suggests involvement of these regions in the initiation of the SE by the systemic injection of pilocarpine.

Previously, two MRI studies have also shown a decrease of brain perfusion in epilepsy-related areas during SE induced by pilocarpine in rats (Choy et al. 2010; Engelhorn et al. 2005). However, an initial cortical activation directly after SE onset was observed in both MRI-based studies, while we found hypo-perfusion in cortical regions directly after the first convulsive seizure and generalized perfusion reduction 24 h after SE. Differently to these published studies (Choy et al. 2010; Engelhorn et al. 2005), anesthesia is not influencing our perfusion imaging as ^99m^Tc-HMPAO uptake is taking place in awake animals. As the tracer gets trapped, animals can be later anesthetized for the scan without influencing uptake. Anesthesia can change systemic perfusion as it inhibits convulsions during SE. In addition, data of these studies were analyzed as ratio between brain regions while we show here absolute uptake values normalized to the injected radiotracer dose. As our data show that e.g. the cerebellum, which is often used as a reference region, shows distinct hypoperfusion, normalizing to this region might lead to virtually increased perfusion ratios in other brain regions. Thus, differences to earlier studies may be due to differences in the SE manifestation due to the use of anesthesia and image analysis approach (Slupe and Kirsch 2018).

Twenty-four hours after SE, we observed a generalized decrease of cerebral perfusion, mainly affecting cortical regions. Cortical regions have shown neuronal loss already hours after SE (Fabene et al. 2007). This early neuronal loss may be associated with the reduction of blood flow acutely after SE. In addition, we have previously shown early reduction in neurotransmitter receptor density and amino acid metabolism 24–48 h after SE (Bascuñana et al. 2018; Bascunana et al. 2019), which may be partially unchained by the hypo-perfusion-induced neuronal death as seen by ^99m^Tc-HMPAO SPECT results obtained from the present study. In addition, chronic epileptic animals also showed hypoperfusion localized in cortical and hippocampal regions. These areas have been described to be affected by neuronal loss and atrophy (Bankstahl et al. 2012; Peredery et al. 2000) together with an enlargement of the ventricles (Niessen et al. 2005) at this time point in the rat pilocarpine model. We used a standard MRI template based on naive Sprague Dawley rats. Thus, hippocampus and cortical ROIs might include ventricle areas in chronic epileptic animals, driving this reduction of ^99m^Tc-HMPAO.

Excess of glutamate release not only participates in the convulsant activity induced by pilocarpine (Costa et al. 2004), but also in seizure-mediated brain injury, which in turn is triggered by intracellular calcium-dependent cytotoxic processes (Revah et al. 2016). Decreases in glucose metabolism have repeatedly found both in epilepsy patients and experimental animal models. Decrease of brain glucose and amino acid metabolism has been frequently attributed to the aforementioned excitotoxicity-mediated neurodegeneration. Nevertheless, reduction of brain perfusion as described in the present study can make an important contribution to such interictal hypometabolic state. Calcium elevation after seizure also occurs in vascular smooth muscle cells and correlates with brain vasoconstriction (Farrell et al. 2017). Thus, regional hypoperfusion/hypoxia due to enduring vasoconstriction together with excitotoxicity and a state of high-energy requirements may account for the seizure-induced neurological impairment (Farrell et al. 2017).

In conclusion, SPECT shows that SE induces brain regional hypoperfusion in rats during at least 24 h. We hypothesized that this reduction in brain perfusion together with excitotoxicity and an elevated energy requirements state may lead to neuronal damage and neuroinflammation as seen in this model. Our results suggest that counteracting perfusion deficiency during SE might serve as a promising pharmacological target to ameliorate SE sequels.

## Data Availability

The datasets used and/or analysed during the current study are available from the corresponding author on reasonable request.
